# Endoscopic nasopharyngectomy for recurrent or persistent nasopharyngeal carcinoma: a 10-year outcome from a tertiary centre in Hong Kong

**DOI:** 10.3389/fonc.2026.1938802

**Published:** 2026-08-26

**Authors:** Ting Cai, Calvin Chee Fung Lai, David Chun Man Yeung, Samuel Man Wai Chow

**Affiliations:** 1 Department of Ear, Nose and Throat, Prince of Wales Hospital, Hong Kong, Hong Kong SAR, China; 2 Department of Otorhinolaryngology, Head and Neck Surgery, Faculty of Medicine, The University of Hong Kong, Hong Kong, Hong Kong SAR, China

**Keywords:** disease-free survival, endoscopic nasopharyngectomy, head and neck oncology, nasopharyngeal carcinoma, overall survival, skull base

## Abstract

**Aim:**

To analyse the outcomes of endoscopic nasopharyngectomy for recurrent or persistent nasopharyngeal carcinoma (NPC) in Prince of Wales Hospital (PWH), a tertiary hospital in Hong Kong.

**Methods:**

A retrospective review on patients who underwent endoscopic nasopharyngectomy in PWH between 2015 to 2025. Patient characteristics, disease status, operative complications, resection margins, need for adjuvant treatment, recurrence and survival outcomes were analysed.

**Results:**

A total of 42 subjects, including 39 recurrent NPC and 3 persistent NPC, were recruited in this study. For disease status, 33 had T1 disease, 5 had T2 disease, and 4 had T3 disease. Only 1 patient had post-operative haemorrhage requiring tracheostomy with no other major complication noted. Negative margin was achieved in 25 patients, whilst 5 patients had close margin (<1mm) and 12 patients had positive margin in final pathology. The 5-year overall survival rate is 77% and 5-year disease-free survival rate is 60%. Prognostic factor analysis showed that patients with negative surgical margin had better disease-free survival. Postoperative adjuvant radiotherapy for positive or close margin did not provide survival benefits in this cohort.

**Conclusion:**

Endoscopic nasopharyngectomy for recurrent or persistent NPC in our centre is of low complication rate, with comparable survival outcomes to other centres in the literature. Margin status is a significant prognostic factor for disease-free survival.

## Introduction

Nasopharyngeal carcinoma (NPC) is an epithelial malignancy originating in the mucosa of the nasopharynx and is associated with Epstein-Barr Virus (EBV) infection (1, 2). It is a rare malignancy worldwide but an endemic disease in Hong Kong, with around 800 new cases per year (3), posing a heavy healthcare burden to the public health system and causing disabilities to patients and families. The gold standard treatment for primary NPC is radiotherapy, with the addition of chemotherapy for advanced-stage disease (4). The local control rate for primary disease is up to 97%, and the 5-year survival rate of primary NPC reaches 90% in patients with early stages (5).

However, around 20% of patients experienced recurrence, and a small portion of patients have persistent disease after initial definitive treatment (6, 7). Treatment for recurrent NPC remains challenging as reirradiation is associated with significant long-term complications including osteoradionecrosis, carotid pseudoaneurysm and blowout, temporal bone necrosis, dysphagia, and others (8–10). Patients receiving reirradiation suffer from poor quality of life and significant mortality (11). In addition, failure of local control after radiotherapy usually indicates radiation resistance (12).

Salvage surgery for local recurrence or residual disease in the form of nasopharyngectomy has demonstrated similar or better prognosis. A large multicentre randomised controlled study compared the efficacy and safety of endoscopic nasopharyngectomy and intensity modulated radiotherapy (IMRT) in patients with surgically resectable recurrent NPC. The results indicated that overall survival rate in the surgery group was significantly higher than that in the reirradiation group, and complications related to radiotherapy were significantly reduced (7). Although proton and heavy-ion radiotherapy reduce the damage to normal tissue compared to IMRT, no high-quality study has demonstrated better prognosis and fewer complications so far. In addition, as a relatively novel and expensive treatment modality, its limited availability also affects its application.

Salvage surgery is recommended in the guidelines of the Chinese Society of Clinical Oncology and the American Society of Clinical Oncology for recurrent T1 and T2 disease (13, 14). Similarly, the clinical guideline for NPC from European Society of Medical Oncology also recommends endoscopic nasopharyngectomy for rT1 to T3 disease if local recurrence does not invade the carotid artery or extend intracranially (15). The national multidisciplinary guidelines of United Kingdom suggest nasopharyngectomy as the first-line treatment for residual or recurrent disease at the primary site and reirradiation as the second-line treatment (16). In the guidelines of Spain and Malaysia, both surgery and radiotherapy/brachytherapy could be selected for recurrent disease (17).

Conventional open nasopharyngectomy can be performed via anterior, lateral, or inferior approaches and is inevitably associated with facial scars and disturbance of mastication and deglutition functions (18–20). Owing to the development of endoscopic skull base surgery in the past decade, endoscopic nasopharyngectomy is gaining popularity compared to conventional open nasopharyngectomy (21, 22). The transnasal endoscopic approach avoids facial scars, and enhanced optics and magnification enable surgeons to better appreciate skull base anatomy and tumour involvement. Application of powered instruments including KTP laser (23), diode laser (24, 25), harmonic scalpel (26), coblator (27) and diathermy (28, 29) enhance intraoperative haemostasis. A recent systemic review and meta-analysis showed that the overall survival rate after endoscopic surgery was significantly higher than that after open surgery for rT1 to rT3 disease. The overall survival for rT4 disease was similar between endoscopic surgery and open surgery, but endoscopic surgery was associated with fewer complications and a lower local recurrence rate (30, 31).

The aim of the current study was to summarise the demographic and clinical outcomes of those who received endoscopic nasopharyngectomy from 2015 to 2025 in a tertiary referral centre in Hong Kong.

## Materials and methods

### Subjects

All consecutive patients with recurrent or persistent NPC who underwent endoscopic nasopharyngectomy at Prince of Wales Hospital, Hong Kong from 2015 to 2025 were retrospectively included in this study. The diagnoses of residual or recurrent NPC was confirmed by pathological examination. Recurrent staging was based on 7th or 8th editions of American Joint Committee of Cancer. A persistent tumour was defined as a residual lesion in the nasopharynx within six months after completion of full-dose radiotherapy with or without chemotherapy and radiotherapy boost on nasopharynx. A recurrent tumour was defined as emergence of new lesions in the nasopharynx at least six months after full-dose radiotherapy, provided that local tumour clearance had been confirmed on a post-treatment biopsy. All patients underwent imaging with contrast-enhanced nasopharyngeal computed tomography (CT) and contrast-enhanced nasopharyngeal and neck magnetic resonance imaging (MRI) as routine preoperative workup. Twenty-eight patients had plasma free EBV DNA checked before operation. Surgery was offered based on the multidisciplinary team discussion involving rhinologists, head and neck surgeons, radiologists, oncologists, and pathologists. The inclusion criteria for surgery were: the presence of recurrent or persistent tumour with no significant contraindication to surgery; for rT1 and rT2 disease, the distance between the tumour and internal carotid canal was more than 0.5cm; and for selective rT3 disease with sphenoid floor or limited clivus involvement after multidisciplinary discussion. Exclusion criteria for surgery were the presence of distant metastases or not fit for surgery. Since 2023, patients with rT3 disease and those with a distance between the tumour and internal carotid canal of less than 0.5cm have been offered neoadjuvant chemotherapy followed by reassessment MRI and multidisciplinary team discussion for operability at our centre.

### Ethical statement

This retrospective chart review study was approved by the Joint Chinese University of Hong Kong - New Territories East Cluster Clinical Research Ethics Committee (Reference number CRE-2026.343).

### Data collection

Demographics, clinical assessment, management regimes, and outcome measures were collected using electronic and paper records. Patient characteristics include age, gender, initial staging, initial treatment modality, time interval between recurrence and the last session of radiotherapy, recurrent staging, and status of lymph node metastasis at the time of recurrence. Clinical management included surgical margins, the use of pedicled flaps to repair the skull base defect, and postoperative adjuvant therapy. Intraoperative clinical outcome measures were collected for this cohort, including operative time, blood loss, and intraoperative complications. Early and late postoperative complications and unplanned readmissions were collected. All patients were followed up regularly at the NPC clinic and rhinology clinic with endoscopic examination. Imaging and EBV DNA were arranged for cases with suspicion of recurrence.

### Salvage endoscopic nasopharyngectomy

**Figure 1:**
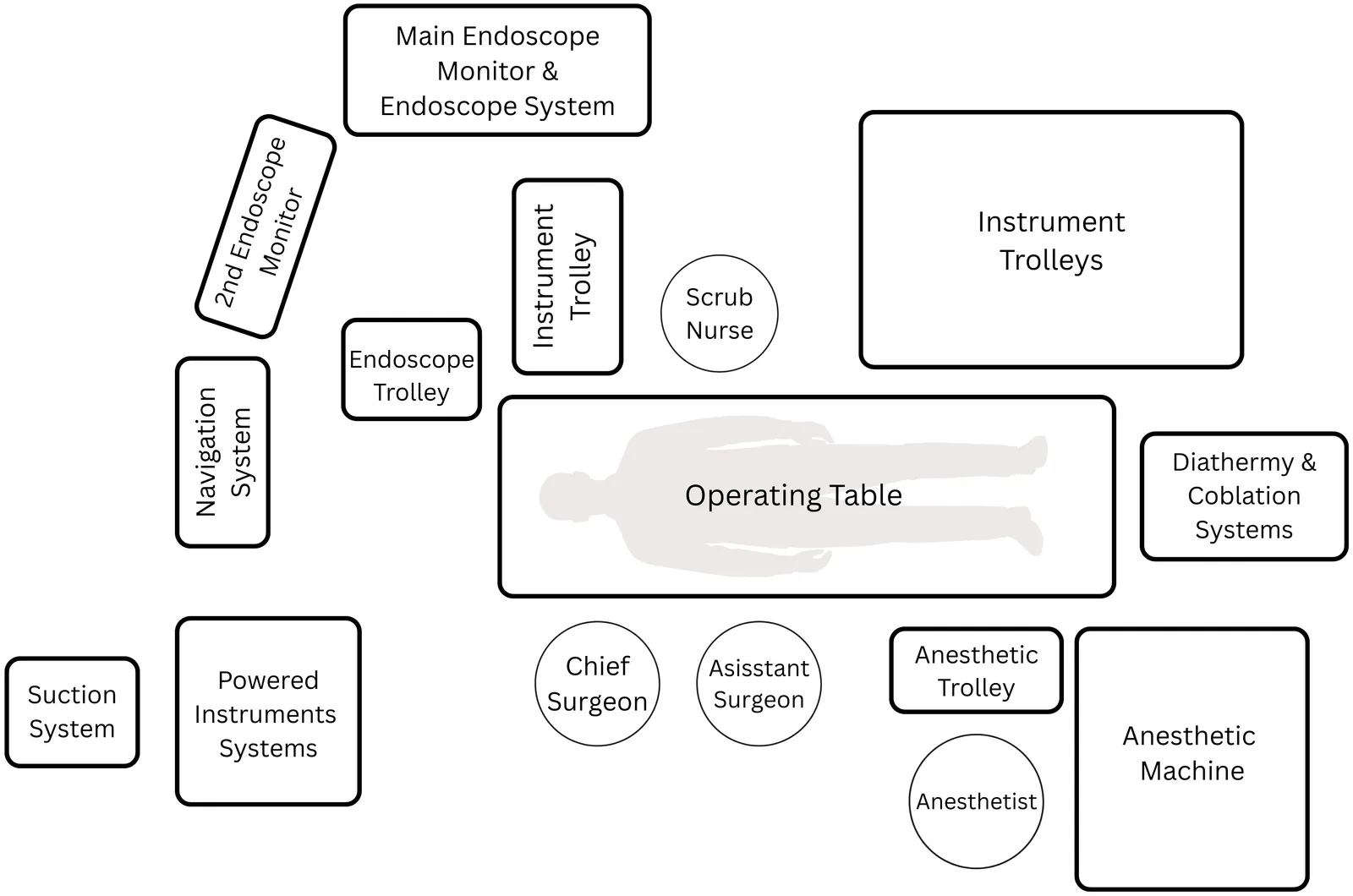
Operating theatre set-up.

**Figure 2:**
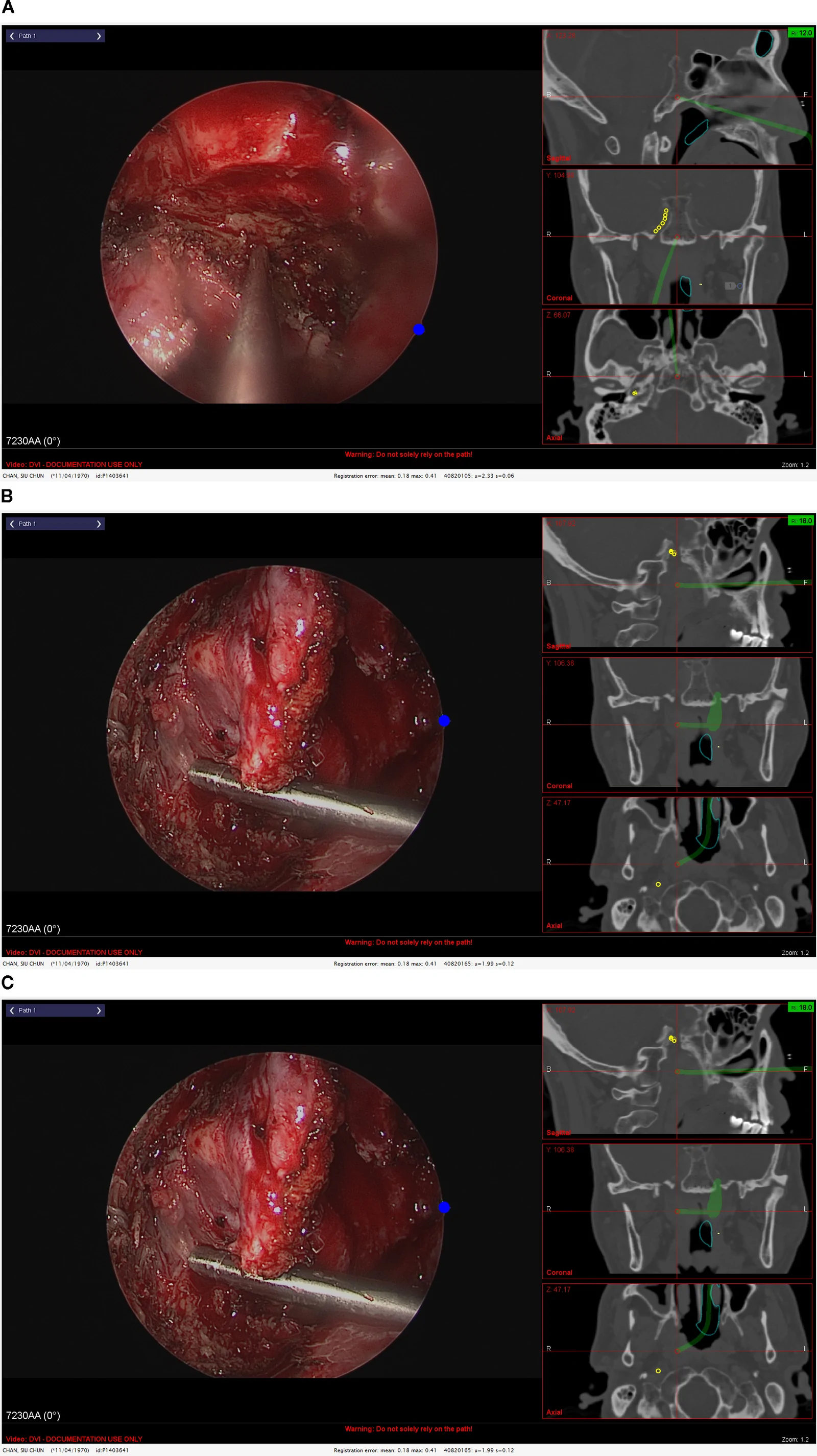
**(A)** Intraoperative navigation with mapping of foramen lacerum. **(B)** Intraoperative navigation with mapping of internal carotid artery. **(C)** Intraoperative navigation showing midline of clivus.

**Figure 3:**
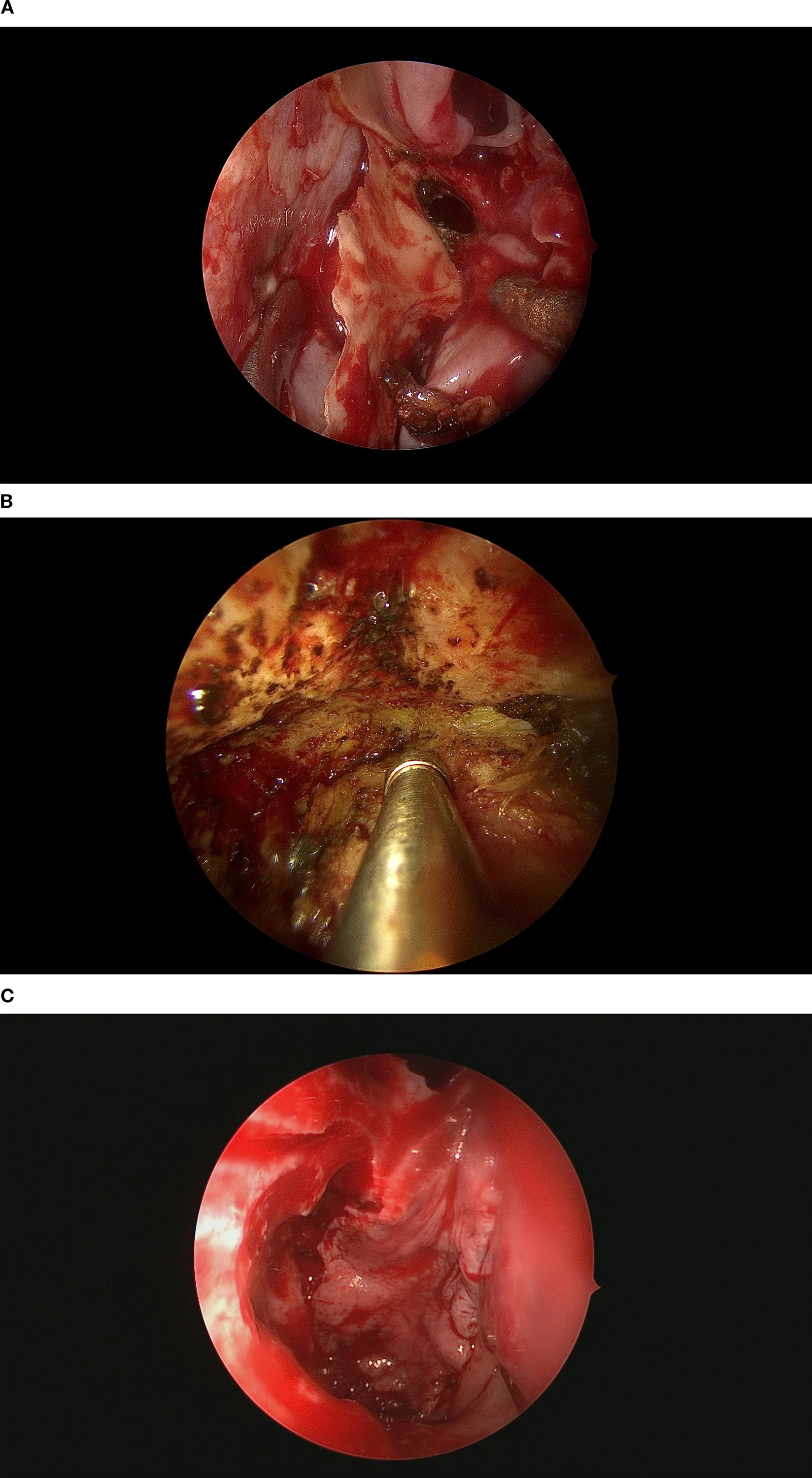
**(A)** Mucosal incision. **(B)** Resection of middle turbinate and medial meatal antrostomy. **(C)** Bilateral sphenoidotomies.

**Figure 4:**
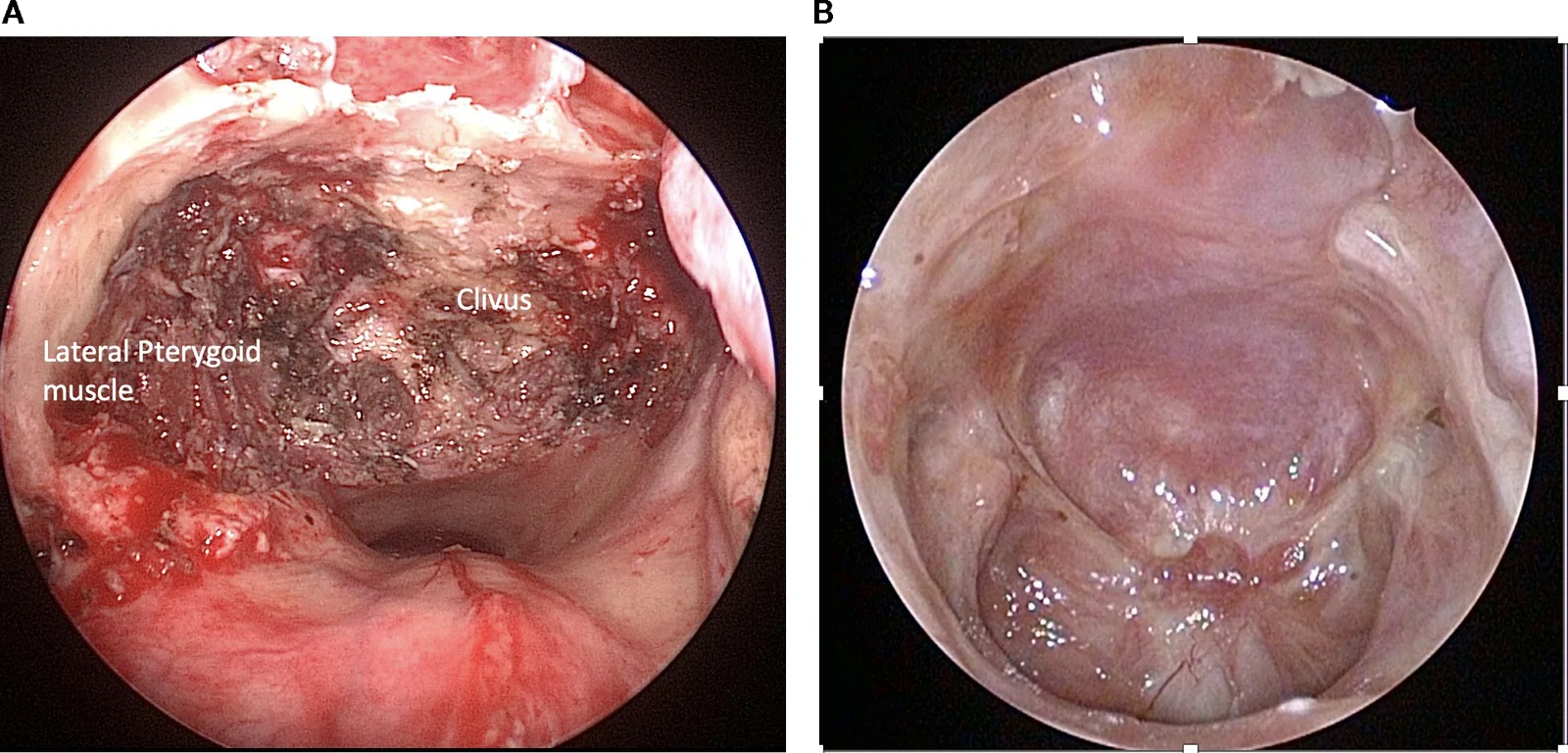
**(A)** Drilling of medial pterygoid plate. **(B)** Clivus as the deep dissection plane.

**Figure 5:**
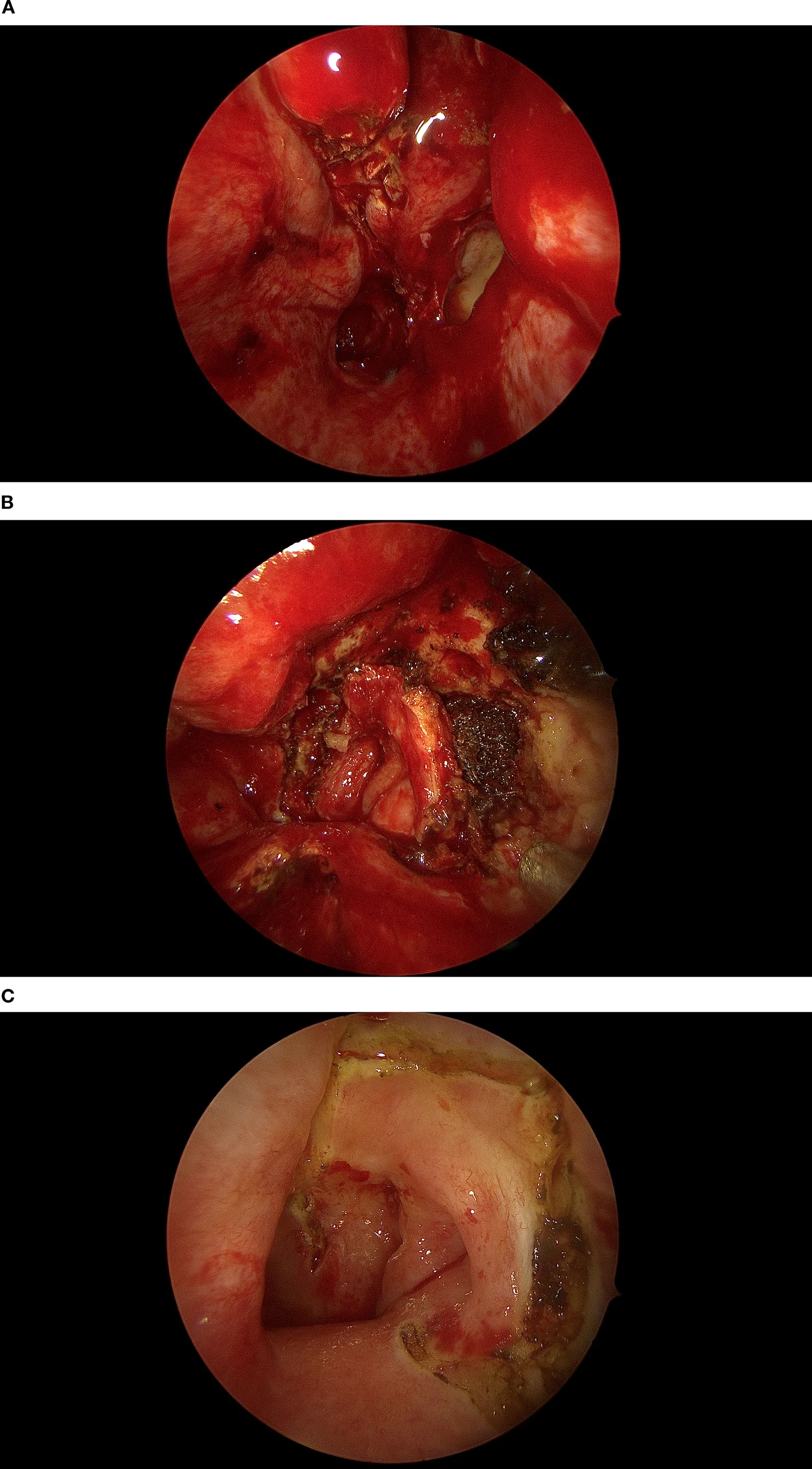
**(A)** Post-excision skull base defect. **(B)** Nasoseptal flap covering skull base defect. **(C)** Nasoseptal flap upon follow-up.

All patients underwent general anaesthesia and were put in a reverse Trendelenburg position with head ring support. The operating theatre set-up is depicted in **Figure 1**. The nasal mucosa was decongested with ribbon gauzes soaked with 3ml 5% cocaine and 3ml 1:10000 adrenaline. Navigation guidance was set up with internal carotid arteries mapped and marked as danger zone (**Figure 2**). 1 cm circumferential mucosal margins were marked with monopolar diathermy around the tumor (**Figure 3A**). Strips of mucosa were taken beyond the resection margins and sent for frozen sections to ensure negative mucosal resection margins. Partial ipsilateral inferior and middle turbinectomy and posterior septectomy were performed for tumor exposure and facilitating bi-nostril four-hand instrumentation (**Figure 3B**). Bilateral wide sphenoidotomies were performed and intersinus septum, sphenoid floor and sphenoid rostrum were drilled down using a 5mm 15-degree diamond burr (**Figure 3C**). Contralateral nasoseptal flap (NSF) was harvested and stored in the newly created common sphenoid sinus cavity for coverage of the nasopharyngeal defect. Coblation assisted nasopharyngectgomy was performed using 2 nostrils 4 hand technique and the ipsilateral cartilaginous Eustachian Tube with the surrounding muscles were resected en bloc with the tumor in all cases. Medial pterygoid plate was removed or drilled down using diamond burr to facilitate lateral dissection (**Figure 4A**). The extent of lateral and deep tissue dissection depended on the extent of disease on the preoperative MRI, aiming at negative resection margins without injury to the internal carotid artery. If the prevertebral fascia was not involved, deep dissection plane was deep to the prevertebral fascia and part of the prevertebral muscle was resected. If the prevertebral fascia was involved without bony erosion, deep dissection plane was down to the clivus (**Figure 4B**). For those selective rT3 cases with sphenoid floor erosion or limited clival involvement, drilling out of diseased sphenoid floor and clivus was performed. Multiple biopsies were taken from the nasopharyngeal defect and sent for frozen section to ensure negative deep resection margins. The nasopharyngeal defect was covered by the NSF (**Figures 5A, B**). If NSF was not available due to previous surgery or disease involvement, lateral nasal wall flap or extended inferior turbinate flap was used instead. The flap position was secured by placing Surgicel pieces on the flap-mucosal edges, overlaid with Tisseel then Nasopore (Self-absorbable) packing. Both nasal cavities were packed with Merocel nasal packing which were kept for 5 days. Patients would be discharged on postoperative Day 6 with their first post-operative outpatient visit at 1–2 weeks upon discharge (**Figure 5C**).

### Statistical analysis

SPSS (version 27, IBM) was used to perform statistical analysis. Continuous data were presented as mean and standard deviation for normal distribution and median and range for non-normal distribution data. Categorical data were reported as frequencies and percentages. Patients’ follow-up was reported in months. Recurrence was calculated from the date of surgery to the date when a mass in the nasopharynx was detected on nasoendoscopy or MRI scan on follow-up visits. Kaplan-Meier product-limit analysis was used to calculate the overall survival (OS) rate and disease-free survival (DFS) rate. OS and DFS were censored at the date of the last follow-up. The log-rank test was used for univariate analysis of potential prognostic factors. A p value < 0.05 was considered statistically significant.

## Results

### Demographics

Thirty-nine patients with recurrent NPC and three patients with persistent NPC were included in this study. **Table 1** displays the demographic, clinicopathological, and treatment modality data of the patient cohort. There were 32 male patients and 10 female patients. The mean age was 56.7 years (range of 26–85 years). Thirty-one patients were aged more than 50 years and 11 patients were aged below or equal to 50 years. Regarding initial T staging, 13 patients had T1 disease, 6 had T2 disease, 19 patients had T3 disease, 2 had T4 disease, and 2 patients had unknown T staging as they were managed in private hospitals for early-stage diseases. Regarding the primary treatment, 8 patients received radiotherapy, 23 patients received concurrent chemoradiotherapy, 6 patients received neoadjuvant chemotherapy followed by chemoradiotherapy, 2 patients received chemoradiotherapy plus an additional neck boost radiotherapy for persistent cervical lymph nodes, and 1 patient received chemoradiotherapy and tympanotomy due to tumour in the middle ear. Two patients with residual local disease received chemoradiotherapy and nasopharyngeal boost radiotherapy. Twenty-eight patients had plasma free EBV DNA checked before operation. Amongst those 28 patients, plasma free EBV DNA was not detected or below 20 in 14 patients. The median time from diagnosis to recurrence was 3 years (range: 1–25 years). For recurrent T staging, the number of patients with T1, T2, and T3 disease were 33, 5, and 4 respectively. Three patients received neoadjuvant chemotheraphy for 3 cycles due to tumor proximity to the internal carotid artery (ICA). Eight patients had simultaneous nodal recurrence; in addition to endoscopic nasopharyngectomy, selective neck dissection was performed in 3 patients, modified radical neck dissection in 4 patients, and parotidectomy in 1 patient in the same operative session for regional control. None of the patients required tracheostomy. The median operative time was 358.5 minutes (range: 155–750 minutes). The median intraoperative blood loss was 250 ml (range: 50-800ml). A pedicled flap was used in all patients for reconstruction of nasopharyngeal defects: a nasoseptal flap was used in 39 cases, and an inferior turbinate flap was used in 3 cases. Length of hospital stay ranged from 1 to 16 days with a median length of stay of 6 days.

**Table 1:** Demographic, clinicopathological characteristics and treatment modalities.

Characteristics	N	%	Characteristics	N	%
Disease status			Disease free interval (months)		
Recurrent NPC	39	92.9	Median (range)	31 (2-131)	
Persistent NPC	3	7.1			
Sex			Recurrent T staging		
Male	32	76.2	T1	33	78.6
Female	10	23.8	T2	5	11.0
			T3	4	9.5
Age (years)			Recurrent N staging		
Median (range)	56.7 (26-85)		N0	34	81
			N1	8	19
Primary T staging			Operative time (minutes)		
T1	13	31	Median (range)	358.5 (155 - 750)	
T2	6	14.3			
T3	19	45.2			
T4	2	4.8			
Unknown	2	4.8			
Primary N staging			Blood loss (ml)		
N0	5	11.9	Median (range)	250 (50-800)	
N1	9	21.4			
N2	16	38			
N3	10	23.8			
Unknown	2	4.8			
Primary treatment			Adjunct procedure		
RT alone	8	19	Neoadjuvant CT	3	7.1
CRT	23	54.8	Selective neck dissection	3	7.1
Neoadjuvant CT + CRT	6	14.3	Radical neck dissection	4	9.5
CRT + neck boost	2	4.8	Parotidectomy	1	2.4
CRT + nasopharyngeal boost	2	4.8			
CRT + tympanotomy	1	2.4			
Plasma free EBV DNA	28	66.7	Skull base resurfacing		
< 20	14	33.3	Nasoseptal flap	39	92.9
> 20	14	33.3	Inferior turbinate flap	3	7.1
Time to recurrence (years)			Length of hospital stay (days)		
Median (range)	3 (1-25)		Median (range)	6 (1-16)	

RT, radiotherapy; CRT, chemoradiothearpy; CT, chemotherapy.

### Safety outcomes

No patient died during the perioperative period. There was no intraoperative ICA injury and no postoperative carotid blowout due to ICA pseudoaneurysm formation. One patient had epistaxis requiring repacking and one patient had persistent bleeding from the anterior ethmoidal artery requiring re-operation for haemostasis. Minor complications such as crusting usually resolved within the first few weeks after nasal irrigation. Twenty patients developed otitis media with effusion. Partial flap loss was noticed in 8 patients; 2 of them underwent another operation for flap coverage, whilst the other 6 patients healed with secondary intention (**Table 2**).

**Table 2:** Safety and oncological outcomes.

Characteristics	N	%
Perioperative complications		
Carotid blowout	0	0
Epistaxis requiring repacking	1	2.4
AEA injury	1	2.4
Post-operative complications		
OME	20	47.6
Partial flap loss	8	19.0
Margin status		
Negative margin	25	59.5
Close margin (<1mm)	5	11.9
Positive margin	12	28.6
Adjuvant treatment		
RT or brachytherapy	4	9.2
CRT	1 (synchronous tongue cancer with positive margin)	2.4
Refused or not offered	12	28.6
Recurrence after surgery		
Non	31	73.8
Local	6	14.3
Regional	2	4.8
Systemic	3	7.1
Survival status		
No evidence of disease	29	69
Alive with disease	6	14.3
Death of disease	2	4.8
Death of other causes	5	11.9

AEA, anterior ethmoidal artery; OME, otitis media with effusion; RT, radiotherapy; CRT, chemoradiotherapy.

### Oncological outcomes

Although all cases achieved negative frozen section margins intraoperatively, the margin status were evaluated on the paraffin-fixed main specimen of nasopharyngectomy. Negative margins were achieved in 25 patients, 5 patients had close margins (<1mm), and 12 patients had positive margins on the final specimen. One patient underwent re-excision for positive superior mucosal margin. Adjuvant treatment was offered to patients with positive and close margins or based on multidisciplinary team discussion, 6 patients received adjuvant radiotherapy or brachytherapy, 1 patient received chemoradiotherapy for synchronous squamous cell carcinoma of the tongue and 10 patients refused adjuvant treatment (**Table 2**).

### Survival

**Figure 6:**
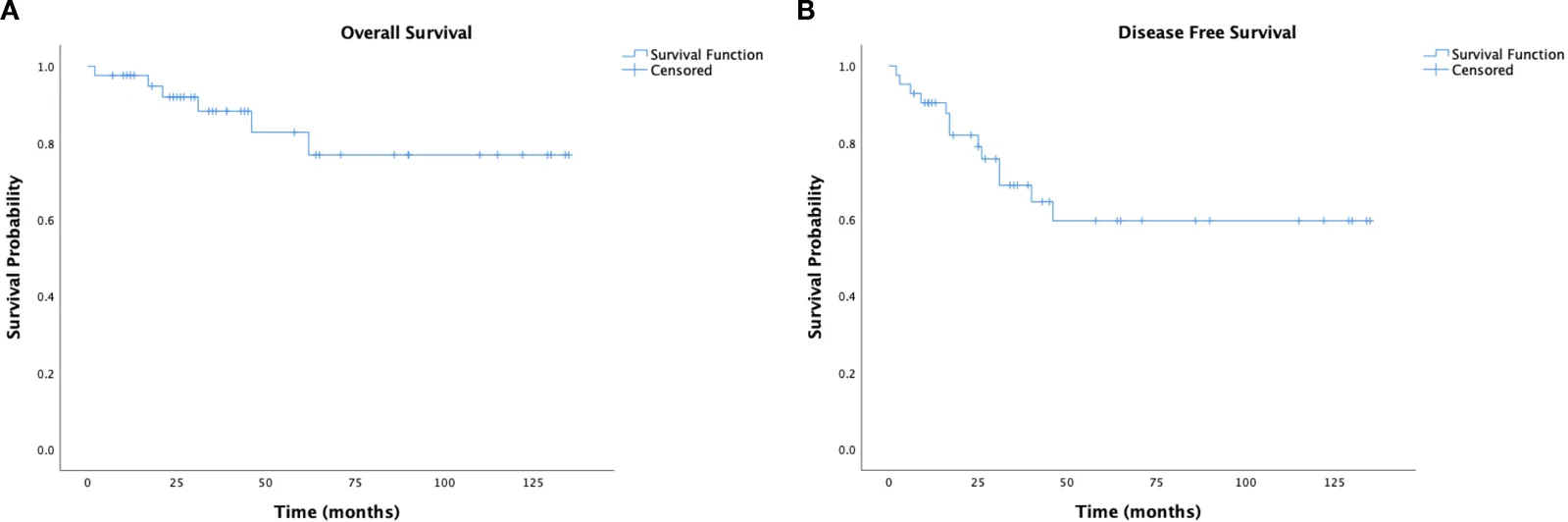
**(A)** Overall survival in all patients. **(B)** Disease-Free survival in all patients.

The median duration of follow-up in the current study was 33 months (range: 2–131 months). Seven patients died during the course of the study. Two patients succumbed to metastatic disease and five patients died of other causes: intracranial haemorrhage in one patient, gastrointestinal bleeding in one patient, and pneumonia in three patients. Twenty-nine patients had no evidence of disease, and six patients were alive with disease. Nine patients experienced recurrence: local recurrence in 6 patients, regional recurrence in 2 patient, and distant metastases in 3 patients. The 2-year and 5-year OS rates for all patients were 89% and 77%, respectively (**Figure 6A**). The 2-year and 5-year DFS rates was 68% and 60%, respectively (**Figure 6B**).

The result of the log-rank test showed no significant difference in OS between patients with different age groups, genders, recurrent T staging, recurrent N staging, margin status, or whether patients received adjuvant radiotherapy (**Table 3**). However, the univariate analysis demonstrated margin status was significantly correlate with prognosis in terms of DFS (p = 0.002), with better survival for patients with negative margins.

**Table 3:** Univariate analysis of overall survival and disease-free survival according to prognostic factors.

Variables	Patient Characteristics	OS			DFS		
		2 year	5 year	P value	2 year	5 year	P value
Gender	Male (32)	88% ± 7%	72% ± 12%	0.599	78% ± 14%	63% ± 18%	0.809
	Female (10)	89% ± 10%	89% ± 10%		64% ± 10%	58% ± 11%	
Age group	< 50 years old (11)	100%	76% ± 15%	0.811	80% ± 13%	68% ± 15%	0.334
	> 50 years old (31)	81% ± 9%	81% ± 9%		64% +/- 10%	56% ± 11%	
Recurrent T staging	T1 (33)	89% ± 6%	81% ± 10%	0.163	69% ± 9%	63% ± 10%	0.157
	T2 (5)	100%	100%		80% ± 18%	80% ± 18%	
	T3 (4)	75% ± 22%	45% ± 27%		50% ± 25%	NA	
Recurrent N staging	N0 (34)	90% ± 6%	77% ± 10%	0.806	65% ± 9%	56% ± 10%	0.384
	N1 (8)	85% ± 14%	85% ± 14%		85% ± 14%	85% ± 14%	
Margin status	Negative margin (25)	85% ± 8%	85% ± 8%	0.584	85% ± 8%	85% ± 8%	0.002
	Close and positive margin (17)	94% ± 6%	59% ± 22%		47% ± 13%	28% ± 13%	
Adjuvant treatment	No (35)	89% ± 6%	89% ± 6%	0.062	72% ± 9%	67% ± 10%	0.235
	Yes (7)	85% ± 14%	39% ± 24%		52% ± 20%	35% ± 20%	

Further subgroup analysis was performed dividing patients based on margin status. Gender, age group, recurrent T staging, recurrent N staging and adjuvant treatment were analysed again in the two subgroups. The results of the log-rank tests are reported in **Table 4**. Recurrent N staging was identified to be significantly correlate with prognosis in terms of OS (p = 0.046) with better survival for absence of neck nodes metastases. However, contrary to our expectation, adjuvant treatment, which was thought to be a protective prognostic factor, was found to be significantly correlated with worse overall survival. Patients who received adjuvant radiotherapy showed worse overall survival compared to patients who did not (p = 0.006). For DFS, there was no significant difference between patients with or without adjuvant radiotherapy (p = 0.400).

**Table 4:** Univariate analysis of overall survival and disease-free survival according to prognostic factors in patients with negative margins and close or positive margins.

Margin status	Variables	Patient Characteristics	OS			DFS		
			2 year	5 year	P value	2 year	5 year	P value
Negative margin (25)	Gender	Male (19)	85% ± 10%	85% ± 10%	0.689	86% ± 9%	86% ± 9%	0.658
		Female (6)	82% ± 16%	82% ± 16%		82% ± 16%	82% ± 16%	
	Age group	< 50 years old (8)	100%	100%	0.158	100%	100%	0.173
		> 50 years old (17)	76% ± 13%	76% ± 13%		76% ± 12%	76% ± 12%	
	Recurrent T staging	T1 (23)	84% ± 9%	84% ± 9%	0.604	84% ± 9%	84% ± 9%	0.615
		T2 (2)	100%	NA		100%	NA	
	Recurrent N staging	N0 (23)	83% ± 9%	83% ± 9%	0.495	83% ± 9%	83% ± 9%	0.507
		N1 (2)	100%	100%		100%	100%	
	Adjuvant treatment	No (23)	83% ± 9%	83% ± 9%	0.553	84% ± 9%	84% ± 9%	0.562
		Yes (2)	100%	100%		100%	100%	
Positive and close margins (17)	Gender	Male (13)	92% ± 8%	37% ± 27%	0.178	36% ± 15%	NA	0.389
		Female (4)	100%	100%		75% ± 22%	45% ± 27%	
	Age group	< 50 years old (3)	100%	0%	0.107	33% ± 27%	NA	
		> 50 years old (14)	92% ± 7%	92% ± 7%		51% ± 15	39% ± 16%	
	Recurrent T staging	T1 (10)	100%	50% ± 35%	0.295	35% ± 18%	NA	0.614
		T2 (3)	100%	100%		67% ± 27%	67% ± 27%	
		T3 (4)	75% ± 22%	45% ± 27%		50% ± 25%	NA	
	Recurrent N staging	N0 (12)	100%	62% ± 23%	**0.046**	42% ± 14%	23% ± 13%	0.455
		N1 (5)	75% ± 22%	NA		75% ± 22%	NA	
	Adjuvant treatment	No (12)	100%	100%	**0.006**	55% ± 15%	41% ± 16%	0.400
		Yes (5)	78% ± 20%	0%		28% ± 23%	NA	

Bolded = statistically significant.

## Discussion

The results derived from the present study support the safety and effectiveness of endoscopic nasopharyngectomy for recurrent or residual NPC. Careful patient selection and adequate surgical resection are essential for achieving favourable oncological outcomes, whilst proper skull base reconstruction helps prevent intracranial infection, life-threatening haemorrhage, and long-term sequelae such as chronic crusting and osteoradionecrosis. Although advances in endoscopic video camera system, image processing technology, surgical navigation system and energy-based devices have improved the safety and efficiency of the procedure, endoscopic nasopharyngectomy remains a high-risk operation because the internal carotid arteries lie within surgical field.

Before the advent of endoscopic surgery, open nasopharyngectomy via the maxillary swing approach was the preferred method for accessing the nasopharynx. It continues to have a role in selected cases, particularly when an endoscopic approach fails or when a free flap is required to cover the exposed internal carotid arteries. Compared with the survival and recurrence outcomes reported for open nasopharyngectomy 15–25 years ago (32–34), our series demonstrated improved oncological outcomes and fewer complications, reflecting the advances in imaging and surgical techniques.

In the available literature, overall survival and disease-free survival appear to be similar in both endemic and non-endemic area. **Table 5** summarises the survival outcomes reported in studies published over the past five years (35–40). The survival rate from the current case series were comparable with the literature however the positive margin rate was higher (28.6%) compared to recent publications. It could be due to the fact that specimen-driven margins were used in our series with measurement of clearance to define positive, close and negative margins whilst most of the literature did not report that. Defect-driven margins were assessed intraoperatively as frozen sections to guide resection area whilst specimen-driven margins were used to evaluate tumour clearance and decide any further resection or postoperative adjuvant radiotherapy is needed. On the other hand, neoadjuvant chemotherapy may also affect margin status. There are three patients with close proximity to ICA who received induction chemotherapy and post-treatment MRI showed shrinkage of tumour. However, deep margins were positive in two patients in subsequent surgeries. It is possible that intraoperative differentiation of tumour with treatment effects and scarring may be obscured by preoperative chemotherapy.

**Table 5:** Summary of survival of endoscopic nasopharyngectomy in the past five years.

Study	Total no. of subjects	T staging	N staging	Positive margins (%)	Defect-driven or specimen-driven margins	Adjuvant treatment	Follow up (mo)	2 year DFS/OS (%)	5 year DFS/OS (%)
Thamboo A, 2021	13	rT1: 9 rT2: 2 rT3: 2	N0: 4 N1: 5	3 (23.1)	NR	RT: 1 CRT: 2	74.3 (56.4-96)	NA	OS 84.6 DFS 53.9
Peng Z, 2021	56	rT1: 9 rT2: 4 rT3: 26 rT4: 17	N0: 33 N+: 23	NR	NR	RT: 4 CT: 5 IT: 3 S: 2 O: 5	44 (1-65)	OS 48.6 DFS 42.6	NA
Wang Z, 2022	37	rT2: 21 rT3: 16	N0: 8 N+: 29	1 (2.7)	Defect driven	RT: 1	31 (5-53)	OS 88.7 DFS 72	NA
Li W, 2021	189	rT1: 55 rT2: 42 rT3: 64 rT4: 28	N0: 144 N+: 45	32 (16.9)	NR	RT: 4 CRT: 7	24 (2-111)	3-year OS 59.5	OS 43.6
Zou X, 2023	56	rT1: 35 rT2: 21		0 (0)	Defect driven	NR		OS 96.4	NA
Valentini M, 2024	41	rT1: 19 rT2: 12 rT3: 8 rT4: 2	N0: 36 N+: 5	7 (17.1)	NR	RT: 5 (positive margins) CT: 4 (positive margins and ECE)	57 (12-139)	NA	OS 60.7 DFS 39.7

RT, radiotherapy; CRT, chemoradiotherapy; CT, chemotherapy; NA, not applicable; NR, not reported; IT: immunotherapy; S, surgery; O, other treatment; ECE, extracapsular extension.

In our case series, most surgical candidates presented with early recurrent T staging. Selective T3 disease with sphenoid floor or clivus involvement was also considered surgically resectable after drilling of the bony skull base following soft tissue resection. Tumour within 5mm distance from ICA was considered high risk for intraoperative ICA injury, postoperative carotid pseudoaneurysm formation and carotid blowout and positive margin status. Hence, endoscopic nasopharyngectomy was not offered for tumours < 5mm to ICA in our centre. Instead, neoadjuvant chemotherapy followed by reassessment MRI and multidisciplinary team discussion for operability are offered to this group of patients.

However, in some centres, patients with ICA involvement undergo preoperative digital subtraction angiography and balloon occlusion testing. If a patient passes the balloon occlusion test, ICA embolisation is performed; if the test is failed, external carotid-internal carotid bypass surgery may be undertaken to permit en bloc resection of the ICA together with the tumour (41). A retrospective case-matched study from the same centre including patients with ICA involvement showed significantly higher 2-year overall survival and progression-free survival rates in patients who received preoperative ICA embolisation than in those who did not. ICA involvement was defined as a distance of less than 1.8 mm between tumour and ICA. However, no direct comparison in oncological outcomes exists between endoscopic surgery with pretreatment ICA embolisation and reirradiation with or without chemotherapy (42).

Margin status is one of the most consistently identified prognostic factors in previous studies (43). However, there remains ongoing debate regarding the definition of negative and close margins, as well as the preferred methods used for their assessment, including paraffin-fixed specimen and frozen sections (44). Survival is also influenced by whether postoperative adjuvant treatment is administered. A previous meta-analysis of prognostic factors in salvage nasopharyngectomy for NPC, which included 17 studies, showed that sex, N staging, surgical approach, adjuvant reirradiation and margin status were independent predictors of outcome. Both endoscopic surgery and reirradiation were also independently associated with improved survival (45). A more recent meta-analysis focusing on surgical margins in salvage nasopharynegectomy, which included 12 studies, found that positive surgical margins were associated with significant worse 5-year disease-free survival, although no significant impact was observed on 3-year DFS or OS at 3 years and 5 years (43). In the present series, the log-rank prognostic factor analysis demonstrated margin status is significantly related with disease-free survival but not with overall survival. The results highlighted the importance of oncological clearance of disease and also hinted the relative indolent nature of recurrent NPC and recent advances in palliative chemotherapy and immunotherapy in maintaining survival after recurrence (46, 47). In the current study, final pathology on paraffin-fixed specimens was used to assess margin status, whereas intraoperative frozen section margin assessment was negative in all cases. It could be argued that the specimen-based margin assessment underestimates the true disease clearance as specific ablation techniques, such as low temperature plasma radio frequency ablation, can vaporise certain amount of tissue (40).

Unlike in other similar studies, recurrent T staging did not show a significant association with DFS in the present analysis. This may be explained by the fact that most patients in our cohort had T1 disease, with only five and four patients classified as T2 and T3, respectively. The marked skew in stage distribution may also have reduced the representativeness of the results. In addition, the included T2 and T3 tumours were selected based on their distance from the ICA and endoscopic accessibility, therefore, the T2 and T3 cases in this study may not be representative of all T2 and T3 diseases. Furthermore, the T staging we used for recurrent disease is actually designed for primary tumours, which reflects the extent of the primary tumour and its prognostic implications at initial presentation, but it may not be a reliable indicator of prognosis for recurrent or residual disease. In other words, a different T staging system may be needed for local recurrence to better differentiate surgical candidates and inoperable cases.

Recurrent N staging was identified as a borderline significant prognostic factor in term of overall survival (p = 0.046) but not disease free survival (p = 0.455). Despite the relative small sample size of cases with concurrent regional recurrence in the present series, greater overall disease burden is showed to be associated with worse overall survival.

Adjuvant treatment is usually indicated in cases with positive or close margin as recommended by an international guideline for postoperative management of recurrent NPC (44). Although the definition of close margin remains debatable amongst surgeons and oncologists, a clearance of less than 1 mm is generally prompts adjuvant radiotherapy for better local control (44). Our initial analysis did not show a significant survival difference between patients who received adjuvant treatment and those who did not. However, subgroup analysis focusing on patients with positive or close margins showed a significant survival difference between those who received adjuvant treatment and those who did not. By contrast to expectation, patients who refused adjuvant radiotherapy demonstrated better OS and DFS and the difference in overall survival is statistically significant in the current study.

These findings reveal the poor prognosis in patients with positive or close margins, as well as the considerable morbidity of reirradiation and the radioresistance often encountered in recurrent and persistent NPC. The unexpected result-namely, better survival in patients with positive or close margins who refused adjuvant treatment-is very likely due to the relatively small sample size in this highly selected population which need to be analysed with great caution. Given the limited sample size and predominant rT1 disease in this cohort, the statistical power of the prognostic factor analysis is relatively low. On the other hand, there is no strong evidence supporting the survival benefits of adjuvant treatment in patients with positive or close margins and the international guideline for postoperative management of recurrent NPC is based on expert opinion (44). Similar findings-specifically, a lack of survival benefits of adjuvant radiotherapy were reported in a cohort of patients after open nasopharyngectomy (48). Multidisciplinary discussion amongst ENT surgeons, oncologists, pathologists, and radiologists remains essential in treatment decision-making. Further high quality evidence on the survival benefits of postoperative secondary radiotherapy is warranted to illustrate the indication and regime of radiotherapy.

Plasma free EBV DNA has become available in public hospitals in Hong Kong, and its use may facilitate earlier detection of recurrence. Although no significant correlation was observed between preoperative or postoperative plasma free EBV DNA levels and survival, elevated plasma free EBV DNA level was found in all recurrent cases after surgery, before radiological or endoscopic confirmation of disease recurrence. This suggests that plasma free EBV DNA may serve as a useful surrogate marker for detection of disease recurrence.

Our study has several limitations. First, this was a single-centre retrospective study, and the inherent design introduces potential bias. Second, changes in staging systems and treatment modalities over the past decade may have affected the analysis of prognostic factors. Third, the relatively small sample size compared with large series may have reduced the statistical power. It is important to have a consensus on how to assess surgical margin status for NPC patients, taking into account that the extent of endoscopic en bloc resection is always limited by the ICA and the skull base, and to investigate more thoroughly the survival benefits of adjuvant reirradiation in patients with suspected microscopic residual disease after surgery. Despite these limitations, our findings highlight the safety and effectiveness of endoscopic nasopharyngectomy for recurrent NPC.

## Conclusion

Endoscopic nasopharyngectomy for recurrent or persistent NPC is a safe treatment option of low complication rate, with comparable survival outcomes to other centres in the literature. Margin status is a significant prognostic factor for disease-free survival.

## Data Availability

The raw data supporting the conclusions of this article will be made available by the authors, without undue reservation.
